# Supplementary material for: Neuropeptides from a praying mantis: what the loss of pyrokinins and tryptopyrokinins suggests about the endocrine functions of these peptides
Source: PeerJ. 2025 Feb 27;13:e19036. doi: 10.7717/peerj.19036 (PMC11874938; doi:10.7717/peerj.19036)
Supplement: Supplemental Information 2 [file peerj-13-19036-s002.pdf]

Supplementary data

Neuropeptides from a Praying Mantis: What the Loss of Pyrokinins and Tryptopyrokinins suggests about the Functions of these Peptides

Jan A. Veenstra[1],

[1] INCIA, UMR 5287 CNRS, Université de Bordeaux, France

Content

Fig. S1. Conceptual translation and predicted cleavage of neuroeptide precursors	2
Fig. S2. EFLamide receptors	9
Fig. S3. Ilp genes on chromosome 1	10
Fig. S4. *Tenodera* sirps	11
Table S1. Mantodea genome SRAs analyzed for spots coding tryptopyrokinin and receptors	12
Table S2. Mantodea transcriptome SRAs analyzed for spots coding tryptopyrokinin and receptors	13

Fig. S1. Conceptually translated Tenodera sinensis neuropeptide precursors. Predicted signal peptides are in yellow, putative convertase cleavage sites in red, predicted C-terminal amides in magenta and Cysteine residues in black. Parts of the precursor that are expected to end up in the mature peptide are in blueish grey. Note that since the processing sites of the three neuropeptide-like precursors (NPLPs) are difficult to predict, plausible basic amino acid residues have been indicate that might be important for cleavage.

AKH
MAISRVLVMLAMAAITLLAFCEAQVNFTPNWGKRTVLQDGPCKVSTDALMYIYKFIQSEAQKLVDCEKFGGN*

ACP
MARRFSERTLCWLLVALAVLATTWGQVTFSRDWNPGKRSQSEPPCNAAFRSAEEVCKVLVDEFRQIAACEIRSLLRY
QKEFEEKHADMFLERPEGR*

Agatoxin-like
MRTYLVVLACALLLLGQFVLPTVAGPYLEDADEGLEDYSDNNLERLLQDPAQKRSSLIYLFRRACIRRGGNCDHRPK
DCCYNSSCRCNLWGSNCRCQRMGLFQKWGK*

Agatoxin-like, alternative trancript
MRTYLVVLACALLLLGQFVLPTVAGPYLEDADEGLEDYSDNNLERLLQDPAQKRACIRRGGNCDHRPKDCCYNSSCR
CNLWGSNCRCQRMGLFQKWGK*

Allatostatin-A
MTRYLCYLPLLFLHLTHAVSPTPARGAPEEAGSGVVSSGSGSTAGLLLSHLEDPTTDGDNSDLDLMKRLYDFGLGKR
AYSYVSEYKRLPVYNFGLGKRSNKMYGFGLGKRSPASSQMYSFGLGKRDYDDYYQAEDEDEDIVDEEAGAEEDMQDD
DNIDLVDKRAGHMYSFGLGKRARPYSFGLGKRAPQQKMYGFGLGKRSPASSQMYSFGLGKRDYDDYYQAEDEDEDIV
DEEAGAEEDMQDDDNIDLVDKRAGHMYSFGLGKRARPYSFGLGKRAPQQKMYGFGLGKRGGSMYSFGLGKRGEGRLY
SFGLGKRPVGYGGRQSGSRFNFGLGKRSENSDLEDEMEQEAMEKRVVPKAGQRFAFGLGKREVAPGELEAVRMEENK
KNETHHSGNIVKRSLQYAFGLGKRTPSYSDMDDVEEFSRLVRRPYSFGLGKRVPMYNFGIGKRSEH*

Allatostatin-B
MQSGRGALLGFLLMVIVKSSCQEDPLPDQSRSQSANGEQTQDVTGVQVDEDKRAWHDLQGGWGKRGWQDLSGGWGKR
GWQDLSGGWGKRGWQDLNGGWGKRGWQDLNGGWGKRGWQDLNGGWGKRGWQDLNGGWGKRGWQDLNGGWGKRGWQDL
NGGWGKRGWQDLNGGWGKRGWQDLNGGWGKRGWQDLQGGWGKRDMDLEDDDIESEEDDEAVADEVKRAWSSLRAGWG
KRAADWANFRGSWGKRDPGWNNLKGLWGKRGDNWSRLSAAWGKRDS*

Allatostatin-CC
MGIHHHHQQHSCLLYSVLFLLVVCCSSTSLTERHSRSATAASLGADYPDYQSGVRYDEYPVVVPKRTAMLLDRIMVA
LQKAVDEDKSGRTYPSELAEAKMDLQRRGQQKGRVYWRCYFNAVTCFKRK*

Allatostatin-CCC
MTSCTKLVLVLMLAVLGIVVQGKVLAQAGDKDRLLTEPDLVDDDGGIETALINYLFAKQMVNRLRNQMDISDLQRKR
SYWRQCAFNAVSCFGK*

Allatotropin
MRCSLSMCALLAVTLVAVLAMCGAASSSGSGSRGKPRTIRGFKNVALSTARGFGKRDGSADLVDQASPLDRFTESLP
VEWFVEEMRTNPELARIIVHKFVDADQDGELTAEELLRPVY*

Baratin
MATALCLMVLVVTLAAALPNTLLEDAQQAAHSASGIGGGRREELPTAIPLTSRAKSIHQQHLQGIGMGKPSSHNPHY
SAGQHMQYQKENLAHQQEGKTVSQYEKGYQYGVGKAALDKHVENALLKSELYGDPAAVNQYRYYGGANERKSNQQIT

YPSPTKRSFNPMMSYVLPSDELTNSRSRLKRELGFDPEDVLTVLSLWEADHHTNSESHQGINPSMYSYYGVQPQDIY
RPMEEEEEEAPEDEQEELGLDNDDTSQTDGDWLDSPVVQPSVYPHQFRLDRRGGYFYPQTYPQQHAQYQHPSHKREG
SHWGGFAKDKRFMVSRKRQAKADEDIPILTHLLNHPYHDAGLPINRRVVL*

Bursicon-A

MNCILTALFLLPLTYGVQQVTAADECQVTPVIHVLQYPGCVPKPIPSFACTGRCSSYLQVSGSKIWQMERSCMCCQE
SGEREASVSLFCPKAKPGERKFRKVTTKAPLECMCRPCTTVEESAVIPQEIAGYADEGPLSNHFRKSL*

Bursicon-B

MICKTQRVYMWFLTNLLFIVLPQLVICGDEDPACETLPSEIHLIKEEFDELGRLQRTCNGEVGVNKCEGACNSQVQP
SVITPTGFLKECYCCRESFLRERIITLTHCYDPDGMRLTQEGQATMDIKIREPADCKCFKCGDFSR*

Calctionin, transcript A1

MEWRRRAVTLLACLLVVVARTSSREIAQDIMDSHMRHLQENRRTVQLLKNLLEDLDVNMETVQKRTACYISAGMGHS
CDYRDIIGAADEAKYWKQEFIPGKRRRRDNF*

Calctionin, transcript A2

MEWRRRAVTLLACLLVVVARTSSREIAQDIMDRKRTACYISAGMGHSCDYRDIIGAADEAKYWKQEFIPGKRRRRDN
F*

Calctionin, transcript B

MEWRRRAVTLLACLLVVVARTSSREIAQDIMDSKTVERMKKWCANTGSDSCGNMYVPGSGDDEDYLNHGDNPGKRAL
LKRLSAYRNWARLLKCSTNTGDDSCGNGYVPGSGDDDDYLNNGNNPGKRALNVRPARCSTNTGDDSCGNGYVPGSGN
DNDYLNGGDNPGKRASPKLFVLHRLRCSTNTGDDSCGNGYVPGSGDDNDYLNGGGNPGKRAIPNLFGVLQHRPTRCS
TNTGDDSCGNGYVPGSGDDNDYLNGGGNPGKRASHSINFPEDGRYPQASSVKYASLKKFCDVFPMNPACRNLK*

Carausius NPLP

MAATPLLLVLLAALGLGTSHPNVEDNMIPNDEEILRALLQEGKSPRQQGQPEVEDSLGLPSDEESYNNLKAMMSLGA
TRPGSSGTHHFVSSSFPEIDARGFHESVFDGFGDYYPTWNRQKRDPLGINSRGFHDDVFSQDFGTFHTVKRDHKDDG
KTTILQRRKRDTTSEKQAGKKSQDQDDVGDEGTSADKRRPEMDGSGFHGDTFSGGFGDFWTMKKRRLGGSSFHGLDT
FSSGFGDFDTMKKRKPEMGASGFHGDTFSNGFGDFWTMKKDGQHPHLEHKRRPEMDSSGFHGDTFNSGFGDFWTMKK
RDYYGDTFNPGYGDLWSMKKRRPEMDSSGFHGDTFNSGFGDFWTMKKRRPEMDSSGFHGDTFNSGFGDFWTMKKRRP
EMDSSGFHGDTFNSGFGDFWTMKKRRPEMDSSGFHGDTFNSGFGDFWTMKKRRPEMDSSGFHGDTFNSGFGDFWTMK
KRKPEMDSSGFHGDLFNNGFGDFWTMKKRRPEMDSSGFYGDTFGGGFGDFWTMKKRRPEMDSSGFHGDTFNSGFGDF
WPMKKRSTSSKSHSKQATNTQH*

Crustacean cardiactive peptide (CCAP),

MQLKHVIMACTVVVLLALCGLPLASCDDVIVQKREVDPADMERLLDPKRKRPFCNAFTGCGKKRSDESMGTLVELNS
EPAVEELSRQILSEAKLWEAIQEARAEILRRRQDQASLVSLTFHFISNVKLQSK*

CCHamide-1, incomplete

????...SCLSYGHSCWGGHGKRSSSDIQTDIDVKDTQLFSSRAVPPLENQWELQDLSDIHLMQQMDNESRLSVKEQ
ENEDEVPDDLLFIADDNIGLPHKLARRRILYRPERKLEKMKILQTSP*

CCHamide-2

MGCLTASSWVTLALLIVILIVLQTDPCVAKRGCSAFGHSCFGGHGKRSEDEALAQPGIEQELTLQQDEIPATSQLQA
RMGLSTFLRQWLQSYRRTAGELDEK*

CCRFamide

MKKASWVVPAVVLLVLLVMLCGGNTEVDCSLSELRCELMCQLTELTRQCNKCRSRAPVRFGKRSGHEHHHYPPLLPP
PPPLPLDSEGQPVMPYEEKLKICCGQLLEFLLKSTAVAQRK*

CNMamide
MSCWTPLLWTLVTIAALCFEAEAAPEALHRRAGDPNIMPINALRESEELSDQEKMLREDVAALMEYLQQYQQQQQQ
GGEDEQVALDNGQENYPPVQQLPPALQVRLLRLQDGINKDDSKRGSYMTMCHFKICNMGRKRNLRYPWLRR*

Corazonin
MKSMHLRVLMICCVASVALAQTFQYSRGWTNGKKRSMEENPCNKLQAMRWLMTHCPFQFYLPHADVPKSGLPEASEE
PSLDFVERLRRLIPQEPSRR*

CRF-like diuretic hormone
MLTTAAILVLATFVTCGTAYYGGSPVLEAMAEPVSDYQTTSYLLPRLVAKYRAPHPSQGDWESASDPRFYLLTELDR
DASQAATRRVKRTGGGPSLSIVNPLDVLRQRLLLEIARRRMRQSQDQIQANRDFLQSIGKREVANRTDNEIADYIVS
PAVEKSGVPAPETNR*

DH31
MSCHNILLATALLIGAILMLSVTHAAESVPIASHRNNYITDLDADADSEYVLEMLTRLGQTIMRANDLENSKRGLDL
GLSRGFSGSQAAKHLMGLAAANYAGGPGRRRRSPDDIA*

Ecdysis triggering hormone
MAESSMCFWSLRHCLYLVSLLIVLYASEANGDEGPFFLKASKNVPRIGRRSEYDNFFLKASKNVPRIGRRREMAPLT
EGRDWGVWPWFKTADSIPGPSRRSDYYIHEEGETQPISWTTVEKTMEEAPELWKPELWRKMAEEMSGTDTKRGNNEQ
SQA*

Eclosion hormone-1
MEGRKTSALLILAMAAVVLLCVLEQAEGNGLGICIRNCAQCKKMLGPYFEGQLCAEACVKFKGKMIPDCEDVSSIAP
FLNKLE*

Eclosion hormone-2
MYHRTTMYLLLLTMMWCYGICSKEAITVCITNCGQCKQMFGSYFRGPVCAESCIASKGRLLPDCNNPNTLIGFLKRL
C*

EFLamide, last exon only.
..NGEKRFSLINKDNDAIFVKDSDPQRRSISSSGEVKSPSPRSLGSELLGKRYLHGGKHFKQEYELVKKYVVLSEVL
HKLSLLLNYFE*

Elevenin
MSRGCLRSISLPVILLSTVLLYHLAASEPKSVNCRRWVFHPTCRGVAAKRAYSQDSPAIFLVDNDRKGDSNLEEVLG
LYATPQPHSQSQNRAEQQRSNLRMQQPLAPWSAHGDGFKGESVYDWYLGRKRSRDNDVVYDY*

FMRFamide
MLRIVLALVLAAIASTYPTDNPISESPNILLATSDESDAPRDDSSNDALLNTLAAAMEDCESESEEESDEASKTSDE
DMLSVLPIRRCPSRNFLRFNRGRPDNFIRFGRGGREDSNFIRFGRGKSDSNFIRLGRGGGDRGNDNFIRFGRARSSN
FVRFGRSRPDNFIRFGRGRDSNFLRFGRSGSLEEPAPFGSSLQVDEDNNRVGRGGKAGSNFIRLGRAGSSSFIRLGR
DGEDQDEDIQERETRGRNTANFVRFGRRANNGNFLRFGRSSNSGELRRGKLTDRNFIRLGRSGSNMYEDDTNSGPAR
VERSENSRGFIRFGKRRDEEEDEDNKLVRLGRDVEEIQEPPVLPSSDANEDNDNNTSRTRRSIPYPKPEDEAQEGSY
PVIIATSSGGNSGHDDKVDTSSFRYYSPLIPNYILAPELSLLAPLSGAESTTKRARGDGHNRNYIRLG*

Gonadulin
MKTPVFLVSCPLFFAVMVHFTSGMPSEEDSCLRIISRIVIDDCSKWKRSIMLQEVGSVLRQHRSDIHSRSGHFSNKG
PLGELLGVPSHWVDDDLADVRRQYRQTIQHLWAECCSNKKKCSGDMFKGLCK*

Glycoprotein hormone A2
MVPVSWRLQCCSLLLVLVMLLSLVSRNSAREMDVWKRPGCHKVGHTRKISIPDCVEFHITTNACRGYCESWSVPSAI
DTLRVNPHQAITSVGQCCNIMDTEDVEVKVMCLDGARDLIFKSAKSCSCYHCKKD*

Glycoprotein hormone B5
MIPLNNNNNNNQTFGPTTLVLLLVVLATLLGQTSAAMTMQENTLSNTLECHRRLYGYKVSKTDSAGRVCWDVISVMS
CWGRCDSNEISDWRFPYKRSHHPVCLHDRREVKYVDLRNCDEGAEPGTERYEYLEAISCRCMICKSSEASCEGLRYR
GQRSGPFLGGGR*

Insulin-like growth factor (IGF), short transcript
MCSPQMWRSWTVLLVVVTALDLVRGTPLGRRQLCGRELADTLSSICFGRGYNDPFSASPGTEVPMYARSRTTRGVAD
ECCKTGCTWSTLEQYCNPRPPETSRSPQNVLTAEKHTSILRNTLESSLEASSSSSSSSSSSSKRRSSPRMSKKKKDR
KHDPSGKVEVAENVEQNANKIPPVIGTISPAYMRVPIVLLKRKAQDTAQN*

Insulin-like growth factor (IGF), long transcript
MCSPQMWRSWTVLLVVVTALDLVRGTPLGRRQLCGRELADTLSSICFGRGYNDPFSASPGTEVPMYARSRTTRGVAD
ECCKTGCTWSTLEQYCNPRPPETSRSPQNVLTAEKHTSILRNTLESSLEASSSSSSSSSSSSKRRSSPRMSKKKKDR
KHDPSGKVRGHHKKKGRRGNNRCRCRRRRRRGKVEVAENVEQNANKIPPVIGTISPAYMRVPIVLLKRKAQDTAQN*

short IGF-related peptide 1
MKMWKLLVRLMALAAVCLCADVHGELTLMKRDIPQRYCGSNLVNVLQLVCRGNIYVVPDDKRSGSLLHNTVLPEEDD
ANLWRMEERRFPFRSRLSASSLVPRSFRRSKRQGVVQECCYYKGCTLSELSSYCGRR*

short IGF-related peptide 2
MWRTWILCLFTLLLVEAQLDKRSSTAKRYCGRNLIHILQLVCDSNYYSNTSPTIFNQKKSFPDDVWLQILEDNPVGD
DEPEFPFRSKPRASSFAHRVFRRHSRSRGVVDECCYDKGCTINELRGYCGSSR*

short IGF-related peptide 3
MKNFYIFCVAAIFCGALPIFTNVEAESLNSADSVVMGNPIETYCGSSLYIKLESVCNGNFNNKFHQEVTKCKLEPWG
IPCTIDSACCQTPCTERYIAGYCASTF*

short IGF-related peptide 4
MKKFDFLCFVAIFCGAFPIFTNVEADSFHVYPWEKRGDSPRKYCGNFLADILHLVCKGNYYSITGHNAENLSTQKKT
SGENEDSYWLQSLEEPSQEEFPFRSRLNSASMIRHRMFRRNAGSPGIVQECCVKGCTFSELSLYCAFR*

short IGF-related peptide 5
MWSAYIRLVALAALCLCTLAQAQSDLFQIGEKRNTPQKYCGRNLADILHLVCNGFYYPMFKKSADLDYDMNDAYWVE
SAPSPPQEQLPFPYRSRASATTVVNGGFRRRMRGIYDECCRKSCTILELSSYCGKR*

invertebrate Parathyroid hormone
MRTRLVFACSALVVLALLVIIPQTQGRPYRQKRVSDQRLAELETIMALRRMAGKLVSVPVGFGQVDPAKIGRRRRRS
AELLLQELLNAQANDVDDAEDSEAEDDIRELVGPHRPSQPWLSEWNRRVQVSIIYLL*

Ion transport peptide, transcript a
MAKQDNSIATLAKRALVCCLVVSVTTACLVRASPTSKLVIGHPLSKRSFFDIQCKGVYDKSIFARLDRICEDCYNLF
REPQLHSLCRKNCFTTEYFKGCLEALLLQDETEQIQTWIKQLHGAEPGV*

Ion transport peptide, transcript b
MAKQDNSIATLAKRALVCCLVVSVTTACLVRASPTSKLVIGHPLSKRSFFDIQCKGVYDKSIFARLDRICEDCYNLF
REPQLHSLCRSKCFSSRYFKGCLEALLLTEEEEKFSQMVDFLGKK*

Leucokinin
MWLATRGMNLILLLTAALATEEFLFPSRLPAIQEGLQTRLCQTAGVPYCSYNSPGYSDDQIPEPTEVKISTYTSGTG
KPMLLIAKNTDDLIRSENEADEAEPDAGVGELNPVKKDSAFSSWGGKRNENWNDKHNSEDPVVQLIKKKASAFSSWG
GKRASLDADPSEESDDYELFLPVVEPEDEENHVLKKSFSSWGGKRTFSNWGGKRVSNLDKPRRAFSSWGGKRAFSTW
GGKRDPSLHEAFGEAENAEKRTFASWGGKRKFSSWGGKRNALESIDKKAFSSWGGKRQLPCTNCNTVLAPDSYTGSA
NDKNLSIISFSMEDNGQTPEIFTPNKEKDYSRDIHQQNVFQNEKEESELLKALELGATSASPLKDANINTFLQDNMH
HMKKRDGRFSSITKHLTYPSIVVRGRFSSWGGKRAVKPLANRLSKTASPQTLERQYRRGGEFYAWGG*

Myosuppressin
MRNSCMMLIGVLAVVLVACVTAIPPPQCTSNNLEEIPPRVRKFCAALSTIYELSNAMETYLDDRVVRENTPMVDALP
KRQDVDHVFLRFGRRR*

Natalisin
MRPHAFLIIVVAWNHVVHGEETNPSLQEANHENATNSSVIAEKRVARSDLGGQGESEENPPPFWANRGRSLNLNGER
LRRHEPFFVEEPEWLVVEEEAPIPEEEDHHGECEESRKRRTSRLTGVDEAFWPSRGRRSGYKRPTGHDENAGRGAQD
LAGIVKMFHSTKGNLFNAKDSIKSHGEHRRLTNTPSIEEPFWAARGRSLFLEPRNRRSGLMESLEEPFWAARGRRSG
NSARGRRSEAFSGEEPFWAARGRRSDDPRGKRPESFSGEEPFWAARGRRFDGSQKVRRSQTSSGEEPFWAARGRRME
AGLHDSVSGKTPSPSTHNRLAEAGNPLKQAENQISEIARGRRSPYQKDHYVQFSSSEEPFWAARGKRGLLESLSAEE
PFWAARGKKQYPQMNNWWPIQEAAAALAEDNSEDESFWTALENKLLARRSVYDIVPM*

Neuroparsin
MSVRCTSVTLGVAILAMLLIQRCEGGSLLCKPCMGNECNLEPAGNCEHGVERDYCGWKVCAKGPGEHCGGPSDLMGK
CGEGMICTCGKCSGCSLATLECFFSSDQLHCI*

Neuropeptide F-1, transcript a
MMQSSALCWLVILGCLVLPQLAWSKPTDPEQLAAMADTLKYLQELDRYYSQVARPRFGKRSELRTLPEQETAPEESS
ERMWRRFVSRR*

Neuropeptide F-1, transcript b
MMQSSALCWLVILGCLVLPQLAWSKPTDPEQLAAMADTLKYLQELDRYYSQVARPSPRSESGRMHELSKVERALKML
RLQELDRFYSQRTRPRFGKRSELRTLPEQETAPEESSERMWRRFVSRR*

Neuropeptide F-2
MQQSPVILAGVLACLCVVSPCWSDPMAIGNEIHSRPTRPKVFTSPDELKTYLEQLSNFYAIAGRPRFGKRLAEPAMF
NSLSGPSPAAAATAARNFHFRFPGAAPVPSGSVRSDVYQMLFPYDE*

Neuropeptide-like precursor
MSPSPSQLLLAIAVFVFIVSFHKALTEDAGTEDQDADDKRTIGFMARIGAVPIMGKRYVASLARNGELPFLVRKEWH
KKMHPVMSGGRNIAELLNPPAGKRYIGALARSGNLPFATKRSQNEDGALENENEDVETLLKEAIDAGHLWRIELGAL
REKLLDENSVYPLLDFYETLNANGMTENHDEEKRAFPAIEPLGSVPFGRKRSVEALARAGYLPQLKPPQESEEYGQR
DSSEASEELLKRSAAGLSRNGNLKGLFQEILEGKRGGVGSLARNGYLRIGLDHFSGKRGGIGSLARSGTLRQKKFDE
EDNDELEELMKELNYLENYDDIARGLFGQDFSASGSEKRNIGSLARARDFPFKGVIKRIPFDEEEIIQKRNLASVLR
NRFAQQQGKRNLGSFMRSYGSSFVPTKKEDYTELDEQYKRNIGSMAKNWLLPEHIKNSKREVGNTLLYDGKDCSPAG
ADNEGEKKESFLGPQNDVTFHHVHKKSTHSTASDAPSYEAKNSTVSESDNAEQKSKSRNKREAYYSAAAPSEEYPLP
VLQNSDLYDYEDMADLLSGEGAPKKRFLGRIPQMGRNKPRTNSHSGRRRPQSRNI*

Orcokinin, transcipt A
MSPVASCRTVMMLVAASLVLQLAYAVPTQNDGYREYHGPDGEDDNVAHRLDSIAGGAHLIRELERQGHVPRQARGGL
DSLSGITFGGNKRLDSLSGITFGNQKRNFDEIDRAGFNSFVKKNFDEIDRAGFDSFVKRNFDEIDRVGFGSFVKRNT
PLLLARLYDKENN*

Orcokinin, transcript B

MSPVASCRTVMMLVAASLVLQLAYAVPTQNDGYREYHGPDGEDDNVAHRLDSIAGDASKKLQEINKDRWDEDMMKEL
YLTRNENAHSRIDSIGGGNIVRNTGHRRIATRGLDSIGGGNIIGRSLGGGTRTNILPSLDYLGDNHVRELDSIGGGN
IVGRDENSLYPYESLRSVDPIGGGNIVRAVDSIGGGNIVRNLDSLGGGNFVRSLDPIGGGNIVRSIDTIGGGNIVRS
SNFARALDSIGGGNIVRSIDTIGGGNIVRSSNFARALDSIGGGNIVRSVDPIGGGNIVRGLDPIGGGNIVRSSNFAR
ALDSIGGGNIVRSLDPIGGGNIVRSLDPIGGGNIVRSLDPIGGGNIVRSLDPIGGGNIVRDLGSFEGRRYFPLKSKN
KGSSRGH*

Periviscerokinin

MIATAFCFAITMLVLVSKASGTEPVIKHKDRRRNSGLIAFPRIGRSDLDLQFSYPASDFVSRWITVYKRQGEKKRQT
LIPFPRIGRSDDVAEESPVMVVDGAALPRNTWQIDAHNRLINTEMPWALVTFKDYARELGEVEDEDPILGNDNAHYT
GPQEKQ*

Periplaneta NPLP

MELWVRLILSLLMLLSPIQCTDEHPSDSLKTAIEAVSRRQRDLASFDSGYYPSGGLVGPRDELAFVAAPRDFAGDGQ
PENIGYGYQKSISSPSGMFAPPSQLAPVEQGYSTKSKTLENLILDYLGDDLKPDDDAQEIYYPNADIKRSAFRERYQ
NGRLEAMKKRYMGSSFRERTHQGPNENIEQKRGMMTDALIRKMEEDEEDERRDRGDDRNSNSPRYLELLRTMWRKYR
NENPNDIEIEDVSDDDVGEMLNYLRESGAIDEEDVDGIKTEIKKRQHYGNDYDFHMHNAAMGGWGGQGYRKRWNQRL
DGDENQKSSFLYSLKFVSPAANHEAIESLREEDEMVPDEHDKDILRLAAAESNRRDPAAWWLPALERGEAPEELFEA
PSEEEYQRLMLAQQGEHHVLPNRKRMKSNYDVPDILLAPEKRYLYDTAVIRKRFPVTKRSSNYYTSPPLLHHKNFIN
SDISERRKKKDAMGTSITTTDPKVAQELNQLFSSSSSHSESPLPFTATTHAPSTENSTSPNTTTKHNGTSETKHSNS
ASKKSAEQPIAMSREEAPLEIRKKSINWSEYFGIDRRRKKSEESHPVDEEWLVNQYFNTLAHEKPSLFHVDNDFPHT
IMRKGAMMQPFDTRVFDTDIFARNVQHKLESKKNARESNEDSTIDNMDDKLQHIEDQIVNEAVKYTGAHEGTTDSRE
IQEVKDKVLARLAAAYSLEKMRQALAEFKTSLQAQRMSKYNPENRKVEEGDEKHKRVAVKKEKVEDAKDEKDKRREN
EEDDSEEFLNNPVVVQPMSEGYMGKHFEFFNISEEECPIVDGIFNTCLLMGDEVGDHANLLISICILHQICYLCGPE
IGFPSAAACDHFFASEAHTACRGDPGCQHAAHKGMTFIQRERELTDNNCWNTPCIAHYFLHFPAPLPVSASVR*

Pigment dispersing factor

MKHLAALLVILYLVRMSLTSPVQQYEDDRYPTADKELNAVSPRELANWLMQLILHKGEANICTHKRNSEIINSLLGL
PKVLNEAGRK*

Proctolin

MCSRHILLLALVLMALYAATEARYLPTRSQDDRLDRLRELLRDLLESEIERSNVNNYERRMMFKREVPQIAAEQQLA
PVVSA*

Prothoracicotropic hormone

MKAFTVIQAAVLYAVTCCLCTASASLSEELREQSEEGSSPGCIGFCKKSEWLNTLLGSATKGTMNHEANQSAIVLSK
RNQNSSGSSFREQEEASCACQSSMSLVDLGQRTYPRYVTSAVCSNSLCRGYGTPCQSIYYITHVLRSKKAQTNAWAH
LQDVGPDGEITEMAAVVDTNGYPYTLPGNLSRKWKLDAIRVVAACLCMN*

Pyrokinin, *Tenodera sinensis* lacks this gene.

relaxin/dilp7, incomplete N-terminus

???.MLLHVSTVTAICVLIELSDSTSTEQELEELFKTRSNEDWEQAWHQERHARCQDRLLRHLYWACEKDIYRLYRR
NSQDEEDQPPSEDNSRWPFLSVLEAQVFLRDRRGARRRRAATSSITDECCVRTVGCTWEEYAEYCPSNKRFRKFV*

RYamide, alternative version

MKCSSVMLTVTIVASLAVLTSSATQFYASGRYGKRAEMSGPMFWTGSRYGRSSGSGTVAAALPGGGRLGDTVEVAAR
NERFFGGRRYGKRAEMSGPMFWTGSRYGRSSGSGTVAAALPGGGRLGDTVEVAARNERFFGGSRYGKRGVDQDQAAI
ADSPRGVLAVEDEASQVTCLYTGVTNLYRCYNRKENSSSEESVNSERTK*

short Neuropeptide F
MQGFPTIKCCTIALCFLIVAAEFVAGAPSYSDYETGVRDLYELLLQKEALENRLQAQQALAGQTTHEVVRKANRSPS
LRLRFGRRADPLLAAAASPFMEHSAESGIAEN*

SIFamide
MQKSGVATCVLLLVVILLAEVAMAAYKKPPFNGSIFGKRGTVVEYDAAGRALSAMCEIASEACSAWFSQSENK*

SMYamide
MQMGKTMFTFVMMLLLVTLMAQTTSSHRRIPFSGSMYGKRGGDSYDSKIKSISTMCEVAADICTIWFPPTEN*

Sulfakinin
MCAFFALRMLLLLTVGVYLALQHCATAAPSTSEVSAAGTSVQRARVHSFPRVRARLVPLEPSSDLLSDFIIDDEFAD
FNKRQSTDGKREKEFDDYGHMRFGKREQFDDYGHMRFGRSLD*

Tachykinin
MTHWRISALILVTLVFTVALCTPEESPKRAPSGFLGVRGKKSDTSSSSAVSYDFAEKRAPAMGFQGVRGKKDNDLML
DFDTADKRAPAMGFQGVRGKKDYDTDLLDLDYFDKRAPAMGFMGMRGKKADLDDILGKRAPALGFQGMRGKKDDWND
ESDMYKRAPSSGFHGMRGKKDFDDYMNVYPGDKRMGFMGMRGKKKEFDEEDYEEALSNGDFWNEEEYLEGESKRAPA
AGFFGMRGKKGPSSGFFGMRGKKGPSAGFFAMRGKKAPGSGFMGMRGKKAPSAGFMGMRGKKDYEDEGDSLESLLQQ
LGYEQAKGREKRTSGQWSMDQGKTILILSIHRLST*

Trissin
MAGTTAHLIFLVTGLVLCTWSVALSCDSCGRECQAACGTRNFRTCCFNYLRKRSDGNALDRPGLRLELLVVPELAAR
YWEDHLKPLHPVPVFTEPEDTTENTRGHMQLIYNP*

Tryptopyrokinin, *Tenodera sinensis* lacks this gene.

Vasopressin (Inotocin)
MSQSEMKTQWFLMFVTTICISSACLITNCPKGGKRTPYDKQDTIKQCARCGPARLGHCYGPAICCGPQIGCLVATPD
TARCLEEAASPVPCVAPTGPVCGVGDTLGRCTANGVCCTHDSCSLDVSCRITVGDTLELMDGGPMFNLYNRRHSLID
SQ*

```
Locusta         MRPAGAAVALAALLPLLLAAGAAASDAAVAPPAAEPAYYSARYRLVGTLCQGVVLAVGLA    60
Tenodera        ----------------------MMNYSVESLEREDAEYYSYRYRTIGTIFQGIILIVGVL    38
                                        . :. :    :  *** *** :**: **::* **:

Locusta         GNLLVVAVVCGARSMRSPTNCYLVSLAVADCLVLVASVPNEIASYYLVGNQWLWGDAGCA    120
Tenodera        GNVMVVVVVHRTRSMRTPTNCYLVSLAVADCLVLVVTVPQEIASYYLVGNLWLWGKAGCN    98
                **::**.**  :****:******************.:**:********** ****.***

Locusta         AFVFSQNLGINASALSLVAFTVERYVAICRPLRSHALRSVARARRVSLLAWAAAAAYSAP    180
Tenodera        IYVFCQYLGINASSLSLVAFTVERYIAICKPLHAHAVCTVSRAQRIAFGVWIFATLYCSP    158
                 :**.* ******:***********:***:**::**: :*:**:*::: .*  *: *.:*

Locusta         WLLLAATRPLRYRGLPELRACAFRLERARYLPYFLCDLLLFYAAPLLLCCVLYALIARAL    240
Tenodera        WILLSGTSPLLYKGLPDGESCGFKLPRAHYLAYFFTDLVLFYVIPLIVSCVLYSLIAKVL    218
                *:**:.* ** *:***: .:*.*:* **:** **: **:***. **::.****:***:.*

Locusta         FRRAALAASGGAGLSPHASAAGVDARCQVVRMLAAVVAAFAALWLPYRGLLVYNSFATLL    300
Tenodera        RSRRIAGS-------AQQIESTAPAREQVVKMLAVVVVVFATLWLPYRGMLVYNSFATLF    271
                  *   .:        :   : . ** ***:***.**..**:*******:*********:

Locusta         SGDKYMDLWFLLFAKTCVFVNSAINPILYNAMSAKFRRAFRRALLRCTRRAAAAAAPADG    360
Tenodera        SGQRFMNLWFLMFAKTCVYINCAINPILYNAMSVKFRRAFRRTLCGGGISAKDS------    325
                **:::*:****:******::*.***********.********:*      *  :

Locusta         PLSGSGGTRLMV---------------------       372
Tenodera        -SSSRGSAGLLQQKKHPSAHRTHSLSTTLSTKR       357
                  *. *.: *:
```

Locusta: QGT41395.1, coding sequence the *Tenodera* sequence as deduced from the genome:

Tenodera deduced coding sequence of ELLamide receptor.

```
ATGATGAACTATAGTGTGGAGTCGCTGGAGCGTGAAGATGCTGAATATTACTCTTATCGG
TACCGTACCATCGGTACCATCTTCCAGGGCATCATACTCATTGTTGGTGTACTGGGCAAC
GTCATGGTCGTCGTCGTGGTGCACCGTACACGTTCAATGCGCACTCCTACCAACTGTTAC
TTGGTGAGCCTAGCAGTGGCGGATTGCCTCGTGCTCGTGGTGACGGTGCCACAAGAGATA
GCCAGCTACTACCTGGTCGGCAACCTGTGGCTGTGGGGCAAGGCCGGCTGTAACATCTAC
GTGTTTTGTCAATATCTGGGGATCAACGCCTCCTCGCTGAGCCTGGTAGCCTTCACTGTG
GAGCGCTACATCGCCATCTGCAAACCTCTGCACGCTCATGCGGTGTGTACAGTGTCCAGG
GCCCAGCGTATTGCCTTCGGTGTCTGGATATTCGCCACCCTGTACTGCTCGCCCTGGATC
CTGCTCTCCGGCACAAGTCCTCTTCTCTATAAGGGTTTGCCAGACGGCGAGAGCTGTGGT
TTCAAGCTTCCTCGTGCACACTACCTGGCCTATTTCTTCACAGACCTCGTGCTTTTCTAC
GTAATACCTCTCATAGTATCCTGTGTGCTGTACTCTCTGATCGCCAAGGTCCTGCGCAGT
CGTCGAATTGCTGGATCTGCGCAACAAATTGAGTCTACTGCACCAGCCAGAGAACAGGTG
GTGAAGATGCTGGCAGTAGTAGTGGTGGTGTTCGCTACCTTATGGCTGCCATACAGAGGG
ATGCTGGTTTACAACTCCTTCGCTACCTTGTTCTCCGGACAGAGATTCATGAACCTTTGG
TTCCTCATGTTCGCCAAGACGTGTGTTTACATCAACTGTGCCATCAACCCAATCCTGTAC
AATGCTATGTCTGTCAAGTTCAGGAGAGCATTCCGGAGGACGCTGTGTGGAGGAGGAATT
TCAGCGAAAGACAGCAGCAGCAGTCGAGGATCAGCAGGCCTGTTACAGCAGAAGAAGCAC
CCTTCTGCACATCGCACACATTCCTTGTCTACAACACTCTCAACCAAGCGATAG
```

Fig. S2. EFLamide receptors. Sequence alignment of EFLamide receptors from *Locusta migratoria* and *Tenodera sinensis* and the coding sequence of the *Tenodera* receptor.

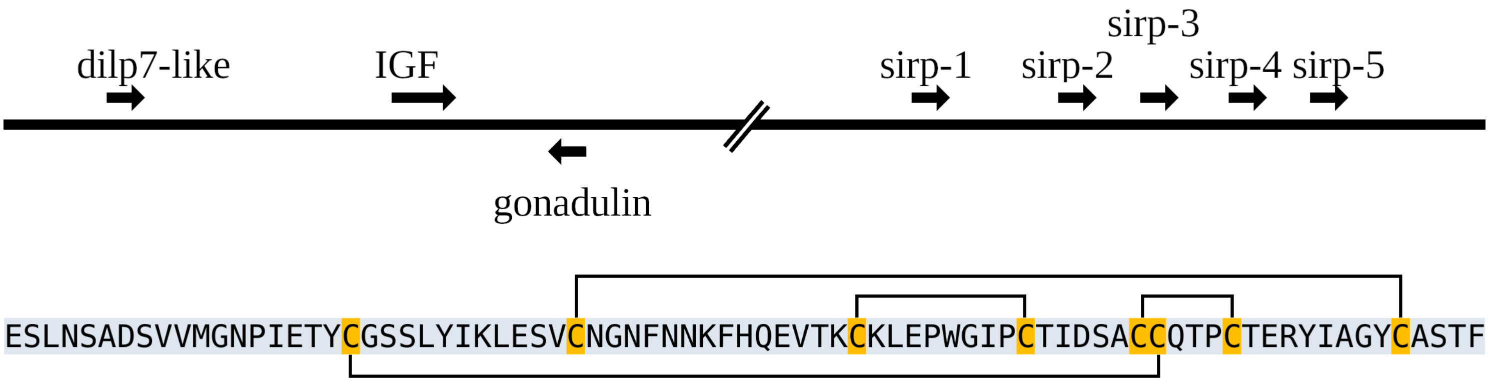

Fig. S3. Ilp genes on chromosome 1. Top schematic representation of the relative orientation of the various *Tenodera* ilp genes on chromosome 1. The dilp7 ortholog, IGF and gonadulin are present in a very similar configuration as in cockroaches. The five sirp genes are locate next to one another on a different fragment of the same chromosome. These two fragments are separated by more than 40,000,000 bp.

```
T.angustipennis-1      MKMWKLLVRLMALAAVCLCADVHGELTLMKRDIPQRYCGSNLVNVLQLVCRGNIYVVPDD     60
T.sinensis-1           MKMWKLLVRLMALAAVCLCADVHGELTLMKRDIPQRYCGSNLVNVLQLVCRGNIYVVPDD     60
                       ************************************************************

T.angustipennis-1      KRSGSLLHNTVLPEEDDANLWRMEERRFPFRSRLSASSLVPRSFRRSKRQGVVQECCYYK     120
T.sinensis-1           KRSGSLLHNTVLPEEDDANLWRMEERRFPFRSRLSASSLVPRSFRRSKRQGVVQECCYYK     120
                       ************************************************************

T.angustipennis-1      GCTLSELSSYCGRR    134
T.sinensis-1           GCTLSELSSYCGRR    134
                       **************


T.angustipennis-2      MANTKMWRTWILCLFTLLLVEAQLDKRSSTAKRYCGRNLIHILQLVCDSNYYSNTSPTIF     60
T.sinensis-2           -----MWRTWILCLFTLLLVEAQLDKRSSTAKRYCGRNLIHILQLVCDSNYYSNTSPTIF     55
                            *******************************************************

T.angustipennis-2      NQKKSFPDDVWLQILEDNPVSDDEPEFPFRSKPRASSFAHRVFRRHSRSRGVVDECCYDK     120
T.sinensis-2           NQKKSFPDDVWLQILEDNPVGDDEPEFPFRSKPRASSFAHRVFRRHSRSRGVVDECCYDK     115
                       ********************.***************************************

T.angustipennis-2      GCTINELRGYCGSSR   135
T.sinensis-2           GCTINELRGYCGSSR   130
                       ***************


T.angustipennis-3      MKNFYIFCVAAIFCGALPISTNVEAGRLNAYAREKRDVIISEYPKIYCGRELNEALQIVC     60
T.sinensis-3           MKNFYIFCVAAIFCGALPIFTNVEAESLNSA----DSVVMGNPIETYCGSSLYIKLESVC     56
                       ******************* *****  **:      .*::.:  : *** .*   *: **

T.angustipennis-3      DGKFNPIPVGGTAARGKRDYDIRRRPRGVVDECCKNPCTREVLEQYCA---   108
T.sinensis-3           NGNFNNKFHQEVTKC---KLEPWGIPCTIDSACCQTPCTERYIAGYCASTF   104
                       :*:**       .:      . :  *  : . **:.***.. :  ***

T.angustipennis-4      MKKFDFFCFVAIFCGAFPIFTNVEADSFHVYPWEKRGDSPRKYCGNFLADILHLVCKGNY     60
T.sinensis-4           MKKFDFLCFVAIFCGAFPIFTNVEADSFHVYPWEKRGDSPRKYCGNFLADILHLVCKGNY     60
                       ******:*****************************************************

T.angustipennis-4      YSITGHNAENLSTQKKTSGENEDSYWLQSLEEPSQEEFPFRSRLNSASMIRHRVFRRNAG     120
T.sinensis-4           YSITGHNAENLSTQKKTSGENEDSYWLQSLEEPSQEEFPFRSRLNSASMIRHRMFRRNAG     120
                       *****************************************************:******

T.angustipennis-4      SPGIVQECCIKGCTFSELSLYCAFR     145
T.sinensis-4           SPGIVQECCVKGCTFSELSLYCAFR     145
                       *********:***************


T.angustipennis-5      MWSAYIRLVALAALCLCMLAQAQSDLFQIGEKRNTPQKYCGRNLADILHLVCNGFYYPMF     60
T.sinensis-5           MWSAYIRLVALAALCLCTLAQAQSDLFQIGEKRNTPQKYCGRNLADILHLVCNGFYYPMF     60
                       ***************** ******************************************

T.angustipennis-5      KKSADLDYDMNDAYWVESAPSPPQEQLPFPYRSRASATTVVNGGFRRRMRGIYDECCRKS     120
T.sinensis-5           KKSADLDYDMNDAYWVESAPSPPQEQLPFPYRSRASATTVVNGGFRRRMRGIYDECCRKS     120
                       ************************************************************

T.angustipennis-5      CTILELSSYCGKR     133
T.sinensis-5           CTILELSSYCGKR     133
```

Fig. S4. *Tenodera* sirps. Sequence comparison of the five short IGF-related peptides from *Tenodera angustipennis* and *T. sinensis*. Note that most of them are well conserved, but that sirp-3 is very different. In *T. sinensis* it has 8 cysteine residues, but in *T. angustipennis* only 6.

SRA	Species	TryptoPK	PeriVKR	TryptoPKR	PKRA	PKRB
SRR18233046	*Amantis wuzhishana*	absent	present	absent	absent	absent
SRR18210554	*Anaxarcha graminea*	absent	present	absent	absent	absent
SRR18218783	*Anaxarcha sinensis*	absent	present	absent	absent	absent
SRR18217670	*Anaxarcha sp.*	absent	present	absent	absent	absent
SRR18215879	*Anaxarcha tianmushanensis*	absent	present	absent	absent	absent
SRR18246376	*Gonypeta brunneri*	absent	present	absent	absent	absent
SRR18246425	*Gonypeta sp.*	absent	present	absent	absent	absent
SRR15584233	*Hierodula chinensis*	absent	present	absent	absent	absent
SRR18217671	*Hierodula latipennis*	absent	present	absent	absent	absent
SRR16641550	*Hierodula longa*	absent	present	absent	absent	absent
SRR16955306	*Hierodula maculata*	absent	present	absent	absent	absent
SRR16955305	*Hierodula sp.*	absent	present	absent	absent	absent
SRR15590770	*Hierodula zhangi*	absent	present	absent	absent	absent
SRR18233043	*Odontomantis sp.*	absent	present	absent	absent	absent
SRR18245581	*Sinomiopteryx sp.*	absent	absent	absent	absent	absent
SRR18212399	*Statilia agresta*	absent	present	absent	absent	absent
SRR16641552	*Statilia flavobrunnea*	absent	present	absent	absent	absent
SRR16641551	*Statilia maculata*	absent	present	absent	absent	absent
SRR16955304	*Statilia sp.*	absent	present	absent	absent	absent
SRR16641549	*Tenodera angustipennis*	absent	present	absent	absent	absent
SRR16641553	*Tenodera aridifolia*	absent	present	absent	absent	absent
SRR18218046	*Tenodera sp.*	absent	present	absent	absent	absent
SRR18218491	*Titanodula formosana*	absent	present	absent	absent	absent

SRA	Species	TryptoPK	PeriVKR	TryptoPKR	PKRA	PKRB
SRR18233328	*Acromantis hesione*	present	present	absent	absent	absent
SRR18210474	*Acromantis japonica*	present	present	absent	absent	absent
SRR18217669	*Arria brevifrons*	present	present	absent	absent	absent
SRR18245254	*Arria pallida*	present	present	absent	absent	absent
SRR18233417	*Arria pura*	present	present	absent	absent	absent
SRR18245238	*Astyliasula major*	present	present	absent	absent	absent
SRR18246373	*Caliris sp.*	present	present	absent	absent	absent
SRR18231900	*Leptomantella sp.*	present	present	present	absent	absent
SRR18210593	*Phyllothelys sinense*	present	present	absent	absent	absent
SRR18215878	*Phyllothelys werneri*	present	present	absent	absent	absent
SRR18237807	*Pseudempusa pinnapavonis*	present	present	absent	absent	absent
SRR18210333	*Sinomiopteryx sp.*	present	present	absent	absent	absent
SRR18210332	*Sinomiopteryx sp.*	present	present	absent	absent	absent
SRR18246465	*Theopompa maculosa*	present	present	absent	absent	absent
SRR18246455	*Theopompa maculosa*	present	present	absent	absent	absent
SRR18210254	*Theopompa ophthalmica*	present	present	absent	absent	absent
SRR18218670	*Theopropus sinecus*	present	present	absent	absent	absent
SRR18232936	*Theopropus sp.*	present	present	absent	absent	absent
SRR18233413	*Theopropus sp.*	present	present	absent	absent	absent

Table S1. Genome SRAs from Mantodea. These SRAs were analyzed for the presence of spots containing coding sequences for tryptopyrokinin (TryptoPK), or orthologous to four *Periplaneta* GPCRs that are the receptors for periviscerokinin (PeriVKR), tryptopyrokinin, the pyrokinin-1 receptor (TryptoPKR), or the two other pyrokinin receptors (PKRA and PKRB). Note that in genome SRAs a GPCR is readily detected, there is only one SRA in which no spots for the periviscerokinin receptor were found. In orange the *Leptomantella* SRA that contains spots for the pyrokinin-1 receptor.

SRA	Species	TryptoPK	PeriVKR	TryptoPKR	PKRA	PKRB
SRR2230505	*Amorphoscelis pulchra*	absent	present	absent	absent	absent
SRR2230519	*Chaeteessa sp.*	absent	present	absent	absent	absent
SRR2230520	*Choeradodis rhombicollis*	absent	absent	absent	absent	absent
SRR2230539	*Eremiaphila braueri*	absent	present	absent	absent	absent
SRR2230561	*Hierodula patellifera*	absent	absent	absent	absent	absent
SRR1185954	*Hymenopus coronatus*	absent	absent	absent	absent	absent
SRR1185955	*Hymenopus coronatus*	absent	absent	absent	absent	absent
SRR11669710	*Leptomantella albella*	absent	absent	absent	absent	absent
SRR921615	*Mantis religiosa*	absent	absent	absent	absent	absent
SRR2230571	*Mantoida sp.*	absent	present	absent	absent	absent
SRR2230587	*Omomantis zebrata*	absent	absent	absent	absent	absent
SRR1811980	*Orthodera novaezealandiae*	absent	present	absent	absent	absent
SRR2230593	*Oxyopsis gracilis*	absent	present	absent	absent	absent
SRR2230598	*Paraoxypilus sp.*	absent	present	absent	absent	absent
SRR2230605	*Phasmomantis sumichrasti*	absent	absent	absent	absent	absent
SRR2230615	*Pseudogalepsus nigricoxa*	absent	present	absent	absent	absent
SRR2230554	*Pseudovates hofmanni*	absent	absent	absent	absent	absent
SRR2230621	*Rhombodera basalis*	absent	absent	absent	absent	absent
SRR2230627	*Sphodromantis lineola*	absent	absent	absent	absent	absent
SRR1811993	*Stagmatoptera biocellata*	absent	present	absent	absent	absent
SRR2230636	*Theopropus elegans*	absent	absent	absent	absent	absent
SRR2230638	*Thesprotia graminis*	absent	present	absent	absent	absent
SRR11729950	*Titanodula formosana*	absent	present	absent	absent	absent

SRA	Species	TryptoPK	PeriVKR	TryptoPKR	PKRA	PKRB
SRR2230497	*Acanthops sp.*	present	present	absent	absent	absent
SRR1811954	*Acontista multicolor*	present	present	absent	absent	absent
SRR2230500	*Acromantis sp.*	present	absent	absent	absent	absent
SRR2230504	*Ameles decolor*	present	absent	absent	absent	absent
SRR1811961	*Brunneria borealis*	present	present	absent	absent	absent
SRR1811963	*Cheddikulama straminea*	present	present	absent	absent	absent
SRR1811965	*Creobroter pictipennis*	present	absent	absent	absent	absent
SRR2230526	*Danuria thunbergi*	present	present	absent	absent	absent
SRR2230528	*Deroplatys lobata*	present	absent	absent	absent	absent
SRR921590	*Empusa pennata*	present	absent	absent	absent	absent
SRR2230536	*Ephestiasula pictipes*	present	absent	absent	absent	absent
SRR2230540	*Euchomenella sp.*	present	absent	absent	absent	absent
SRR2230549	*Gonatista grisea*	present	present	absent	absent	absent
SRR2230556	*Harpagomantis tricolor*	present	present	absent	absent	absent
SRR2230559	*Heterochaeta occidentalis*	present	present	absent	absent	absent
SRR2230562	*Humbertiella sp.*	present	present	absent	absent	absent
SRR2230563	*Idolomantis diabolica*	present	absent	absent	absent	absent
SRR2230568	*Liturgusa sp.*	present	present	absent	absent	absent
SRR921620	*Metallyticus splendidus*	present	absent	present	absent	absent
SRR2230577	*Miomantis binotata*	present	absent	absent	absent	absent
SRR2230584	*Nilomantis floweri*	present	present	absent	absent	absent
SRR2230591	*Orthoderella ornata*	present	present	absent	absent	absent
SRR2230594	*Oxyothespis dumonti*	present	absent	absent	absent	absent
SRR2230601	*Parasphendale sp.*	present	absent	absent	absent	absent
SRR1811985	*Phyllocrania paradoxa*	present	absent	absent	absent	absent
SRR2230606	*Phyllothelys werneri*	present	present	absent	absent	absent
SRR1811986	*Popa spurca*	present	absent	absent	absent	absent
SRR2230614	*Pseudempusa pinnapavonis*	present	present	absent	absent	absent
SRR2230624	*Sibylla pretiosa*	present	absent	absent	absent	absent
SRR2230635	*Telomantis lamperti*	present	present	absent	absent	absent
SRR2230641	*Tropidomantis tenera*	present	present	absent	absent	absent

Table S2. Transcriptome SRAs from Mantodea. For explanation see Table S1. Note that GPCRs are not easily detected as in genome SRAs, but that nevertheless in *Metallyticus splendidus* a spot for a pyrokinin-1 receptor was found.
